# Transposable Elements and Teleost Migratory Behaviour

**DOI:** 10.3390/ijms22020602

**Published:** 2021-01-09

**Authors:** Elisa Carotti, Federica Carducci, Adriana Canapa, Marco Barucca, Samuele Greco, Marco Gerdol, Maria Assunta Biscotti

**Affiliations:** 1 Department of Life and Environmental Sciences, Polytechnic University of Marche, Via Brecce Bianche, 60131 Ancona, Italy; e.carotti@pm.univpm.it (E.C.); f.carducci@univpm.it (F.C.); a.canapa@univpm.it (A.C.); m.a.biscotti@univpm.it (M.A.B.); 2 Department of Life Sciences, University of Trieste, Via L. Giorgieri, 5-34127 Trieste, Italy; SAMUELE.GRECO@phd.units.it (S.G.); mgerdol@units.it (M.G.)

**Keywords:** genome evolution, transposable elements, environmental adaptation, fish

## Abstract

Transposable elements (TEs) represent a considerable fraction of eukaryotic genomes, thereby contributing to genome size, chromosomal rearrangements, and to the generation of new coding genes or regulatory elements. An increasing number of works have reported a link between the genomic abundance of TEs and the adaptation to specific environmental conditions. Diadromy represents a fascinating feature of fish, protagonists of migratory routes between marine and freshwater for reproduction. In this work, we investigated the genomes of 24 fish species, including 15 teleosts with a migratory behaviour. The expected higher relative abundance of DNA transposons in ray-finned fish compared with the other fish groups was not confirmed by the analysis of the dataset considered. The relative contribution of different TE types in migratory ray-finned species did not show clear differences between oceanodromous and potamodromous fish. On the contrary, a remarkable relationship between migratory behaviour and the quantitative difference reported for short interspersed nuclear (retro)elements (SINEs) emerged from the comparison between anadromous and catadromous species, independently from their phylogenetic position. This aspect is likely due to the substantial environmental changes faced by diadromous species during their migratory routes.

## 1. Introduction

Teleosts, comprising more than 32,000 extant species [1], represent an evolutionarily successful and highly diverse group of vertebrates that populate a wide range of both sea water (SW) and freshwater (FW) habitats across the world, from polar to tropical regions [2]. The genome composition of these organisms most certainly represents one of the key factors behind such evolutionary success. Two major events of whole genome duplication (WGD) occurred during the early evolution of vertebrates 500 million years ago, and thus also affected the lineage of actinopterygians. Subsequently, a third duplication (3R) took place, 300 million years ago, in the teleost lineage and specific events (4R) occurred independently only in some lineages [3,4,5]. The genome size of ray-finned fish is characterised by a wide range of variation, with the smallest values found in Tetraodontiformes species (~0.35 Gb) and the highest values in Acipenseriformes (~9.5 Gb). These differences are mostly ascribable to the presence of repetitive DNA [6,7,8]. Indeed, as postulated by the C-value paradox, the complexity of an organism is not related to the amount of DNA, because not all DNA is made up of genes, but it is mostly constituted by intergenic non-coding DNA, and in particular by repetitive DNA [9]. These large genomic regions contain highly repeated sequences that are classified into two main groups: transposable elements (TEs) and tandem repeat elements, with the latter including satellite DNA, minisatellites, and microsatellites [10,11,12]. TEs are DNA sequences capable of replicating, moving, and integrating into new regions of the genome. They are divided into two main classes: (i) retrotransposons (Class I) are able to propagate their copy sequences in the host genome by reverse transcription of an RNA intermediate molecule through a copy-and-paste mechanism; (ii) DNA transposons (Class II) are generally eliminated from their original location in order to be inserted in a different portion of the genome by a cut-and-paste mechanism. The Class I retroelements include long terminal repeat (LTR) retrotransposons and non-LTR retrotransposons, these latter encompassing long interspersed nuclear elements (LINEs) and short interspersed nuclear elements (SINEs) [13]. The structure of LTR retroelements comprises the open reading frames (ORFs) encoding for viral structural proteins (GAG), an aspartic protease (Pol), a reverse transcriptase (RT), an RNase H, and an integrase. LINE retroelements encode a reverse transcriptase and a nuclease necessary for transposition. Conversely, SINE retroelements are non-autonomous and their origins are related to tRNA, 7SL RNA, and 5S RNA. The Class II includes autonomous elements characterised by the presence of terminal inverted repeats and the ability to encode for a transposase. This Class of TEs comprises also *Helitrons* that use a rolling-circle mechanism for replication [13]. Although repetitive DNA was long considered to be devoid of any functional meaning and therefore labelled as “junk DNA”, a large number of reports now support a functional and evolutionary role for a significant fraction of the non-coding portion of vertebrate genomes [14,15,16,17]. In particular, TEs can be considered important factors for the reorganisation of the genome through chromosomal rearrangements such as duplications, inversions, translocations, and creation of novel genes or regulatory elements through molecular domestication [7,18,19] but also function themselves as enhancers, promoters, silencers, and boundary elements [20,21]. Overall, the fish mobilome shows a wide variety of TE superfamilies, and, compared to other vertebrates, it is mainly dominated by DNA transposons [22,23,24,25]. In addition, some lineage-specific TEs have played a significant role in karyotype evolution and, in particular, in the genesis of sex chromosomes [26,27,28,29,30,31]. In plants, several works have highlighted a relationship between TEs and the environment to which species are adapted [32,33,34,35,36]; however, growing evidence suggests that TEs might be among the main drivers of adaptation even in animals [37,38]. This aspect is particularly intriguing in fish, which are characterised by a high biodiversity [39,40,41].

Therefore, the possible correlation between TEs and migration, one of the most intriguing behaviour of fish, is of extreme interest. To cope with aquatic environments characterised by substantial differences in salinity and temperature, migrating organisms have evolved an extraordinary physiological plasticity. In this context, diadromous fish species represent a particularly interesting case study. This group of fish has always attracted scientific attention due to their spectacular migratory routes between SW and FW, and some of them are also of commercial importance for fishing activities. Three different types of diadromy have been recognised: anadromy and catadromy are related to spawning and differ for the passage from SW to FW and vice versa, respectively. A third typology, still debated among fish biologists, is amphidromy, for which migration to SW is not correlated with spawning necessity and occurs in a specific life stage (hatched larvae) for a restricted period of time (up to 200 days), after which juveniles return to FW, where they spend their adult life [42,43]. On the contrary, oceanodromous and potamodromous species migrate within SW and FW, respectively.

While multiple studies are progressively building up a significant amount of evidence corroborating the existence of a correlation between the genomic abundance of particular TE classes and the adaptation to specific environments, to date this fascinating topic has never been comprehensively investigated in migrating fish. With the present study, we aim to fill this knowledge gap by analysing the TE content in 24 fish species, including 15 having a migratory behaviour.

## 2. Results

### 2.1. Evaluation of TE Impact in Bony Fish Genomes

**Figure 1:**
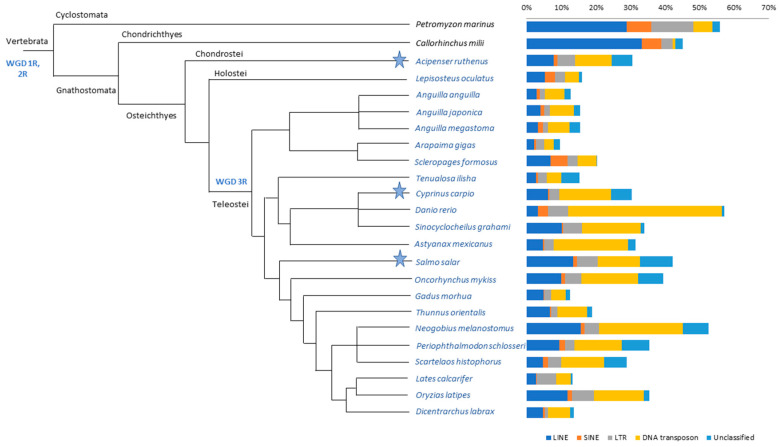
Cladogram showing the relationships between analysed species. Main clades are reported above the branches. 1R, 2R, and 3R whole genome duplication (WGD) events are reported, and the light-blue star indicates species that have undergone 4R-specific events. Species belonging to Actinopterygii are shown in light blue. The cladogram was modified from Betancur-R et al. [44]. The percentages of total transposable elements (TEs) masked in the genomes of the studied species are shown on the right-hand side. Each bar displays the percentage of the main TE types: DNA transposons in yellow; LTR retroelements in grey; SINE retroelements in orange; LINE retroelements in dark blue; and unclassified elements in light blue.

**Figure 2:**
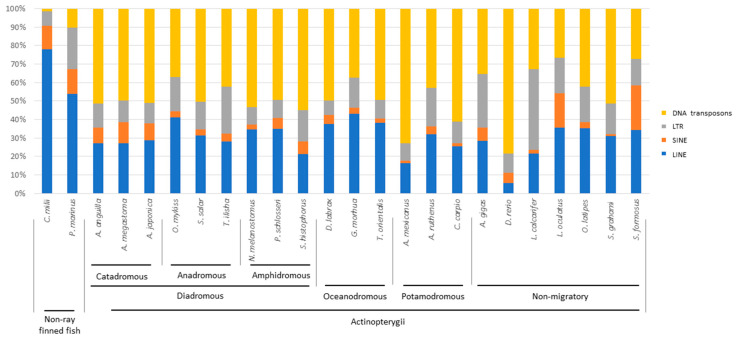
Relative abundance of TE types in the mobilome of the fish species analysed. The histogram shows the relative abundance of each TE type in the mobilome of two non-ray finned fish and 22 actinopterygians. The migratory behaviour of Actinopterygii is indicated.

The masking analysis of genome repetitive fraction showed a variable TE content in fish genomes (Figure 1, Supplementary Table S1). The highest abundances of TEs were detected in the *Petromyzon marinus*, *Callorhinchus milii*, *Neogobius melanostomus*, and *Danio rerio* genomes. The impact of each TE type on the genome of the analysed species was graphically represented as the TE relative contribution (Figure 2), that evidenced a clear difference between the two non-bony vertebrates, where LINE retroelements were clearly prevalent compared to actinopterygians. A balance between Class I retroelements (LINE, SINE, and LTR) and Class II DNA transposons was observed in the three species belonging to the *Anguilla* genus, in *Salmo salar*, *Periophthalmodon schlosseri, Dicentrarchus labrax, Thunnus orientalis,* and *Sinocyclocheilus grahami.* In the remaining ray-finned fish, retroelements were more abundant than DNA transposons, except for *Cyprinus carpio*, *D. rerio*, *Scartelaos histophorus*, *N. melanostomus*, and *Astyanax mexicanus*, where a major impact of DNA transposons emerged. We recorded a very similar amount of DNA transposons and LINE retroelements in *Oncorhynchus mykiss*, *Gadus morhua,* and *Arapaima gigas*. The impact of LTR retroelements on the genome of *A. gigas, Tenualosa ilisha*, and *Lates calcarifer* was higher than the other species. Overall, SINE retroelements had a minor relative contribution in all ray-finned fish species, except for *Lepisosteus oculatus* and *Scleropages formosus*.

We found a positive correlation between TE content and assembled genome size, supported by statistically significant Spearman and Kendall correlations (Spearman: rho = 0.536, *p*-value = 0.009; Kendall: tau = 0.352, *p*-value = 0.019; Supplementary Figure S1).

Moreover, the trends evidenced from the distribution of TE relative abundances was not consistent with species phylogeny.

### 2.2. TE Contribution in Migratory Species Genomes

Nine of the species analysed here were characterised by a diadromous behaviour: the three eel species (*Anguilla anguilla*, *Anguilla japonica*, *Anguilla megastoma*) are catadromous, *T. ilisha*, *S. salar*, and *O. mykiss* are anadromous, while *N. melanostomus, P. schlosseri,* and *S. histophorus* are amphidromous. The comparison of TE relative abundances enabled recognition of distinct patterns in the different fish groups. In particular, concerning retroelements, the three catadromous species presented a higher abundance of LINE, followed by a very similar amount of LTR and SINE retroelements, while SINE retroelements were the least represented in the other six species considered (Figure 2, Supplementary Table S2).

One-way analysis of variance (ANOVA) identified the abundance of SINE retroelements as the only statistically significant difference (*p*-value < 0.05) between anadromous and catadromous species, and the abundance of DNA transposons as the only significant difference between amphidromous and catadromous species.

A potential caveat of this study is linked with the congeneric status of the three catadromous species we considered, which may have led to the introduction of a phylogenetic signature in genomic TE composition in this group. Unfortunately, no fully sequenced genome of other catadromous species not belonging to *Anguilla* genus is presently available to broaden taxonomical sampling.

**Figure 3:**
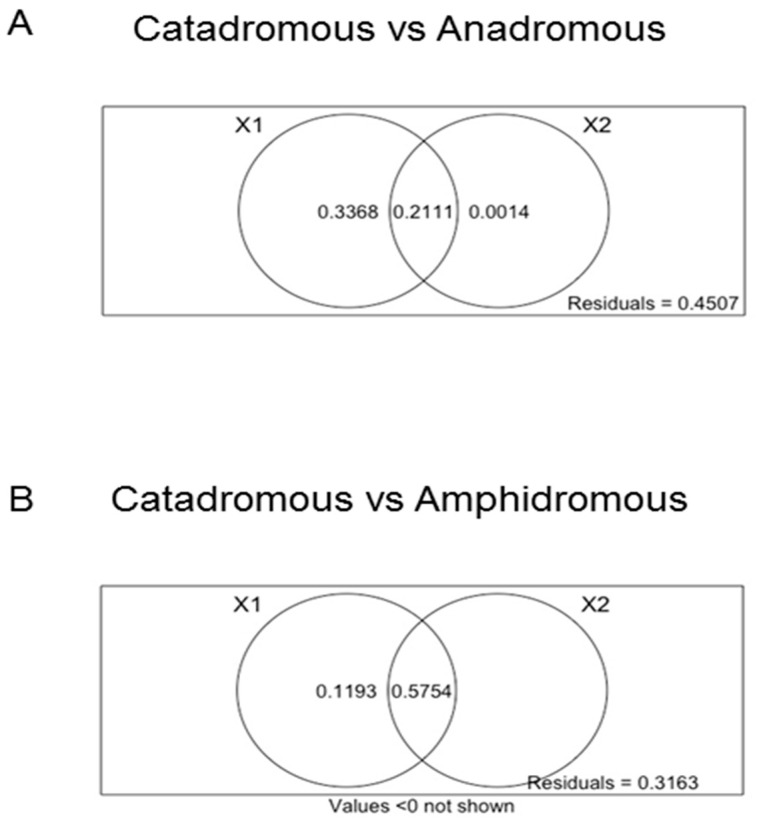
Venn diagrams obtained by variation partitioning analyses (VPA) using redundancy analysis (RDA). The partition of the variation of a response variable (X) between two sets of explanatory variables (X1 and X2) is shown. Each circle represents the portion of variation accounting for an explanatory variable or a combination of the explanatory matrices. The intersection between the two circles represents the amount of variation explained by both variables X1 and X2 [45]. (**A**): the response variable (X) is the quantitative difference reported for SINE retroelements from the comparison between anadromous and catadromous fish species; the explanatory variable X1 represents the migration and the explanatory variable X2 indicates the sequence divergence obtained by p-distance matrix using 16S rDNA. (**B**): the response variable (X) is the quantitative difference reported for DNA transposons from the comparison between catadromous and amphidromous fish species; the explanatory variable X1 represents the migration and the explanatory variable X2 indicates the sequence divergence obtained by p-distance matrix using 16S rDNA.

The variation partitioning analyses were performed to test the influence of phylogeny or migration on TE quantitative composition. Data obtained showed a marked correlation of migratory behaviour on the quantitative difference observed for SINE retroelements in the comparison between catadromous and anadromous species. Moreover, migratory behaviour was found to have a slighter correlation on the quantitative difference of DNA transposons observed between catadromous and amphidromous species (Figure 3).

To provide a fine-scale overview of the expansion of particular TE families in catadromous and anadromous species, we analysed the relative abundance of TE families, generating two Z-score-based heat maps (Supplementary Figure S2). Despite their high phylogenetic distance, the three anadromous species displayed a very similar content of SINE retroelements, much lower than the three closely related catadromous species. Curiously, some elements (*SINE/tRNA* and *SINE/tRNA-Core*) displayed a similar abundance between anadromous and catadromous species, except for *A. anguilla*, where they were particularly expanded (Supplementary Figure S2A). While larger interspecific differences were evidenced by a broader overview of the relative abundance of all TEs (Supplementary Figure S2B), the clustering of anadromous and catadromous species in two distinct groups was still strongly supported. Moreover, the comparison between the two heat maps evidenced distinct relationships between patterns observed for the three species belonging to the *Anguilla* genus: Supplementary Figure S2B shows a cluster comprising *A. anguilla* and *A. japonica,* and in an external position *A. megastoma*, while Supplementary Figure S2A outlines a closer relationship between *A. japonica* and *A. megastoma*; for the anadromous species considered, the two heat maps did not show any difference in terms of relationships between the obtained patterns.

Although a specific pattern was not clear in the comparison between the relative TE abundances of diadromous, oceanodromous and potamodromous teleosts, we observed that oceanodromous species were generally characterised by a higher quantity of LINE retroelements (>37%) compared to potamodromous and diadromous species, except for *O. mykiss* (Figure 2).

### 2.3. Kimura Distance-Based Copy Divergence Analyses of Transposable Elements

**Figure 4:**
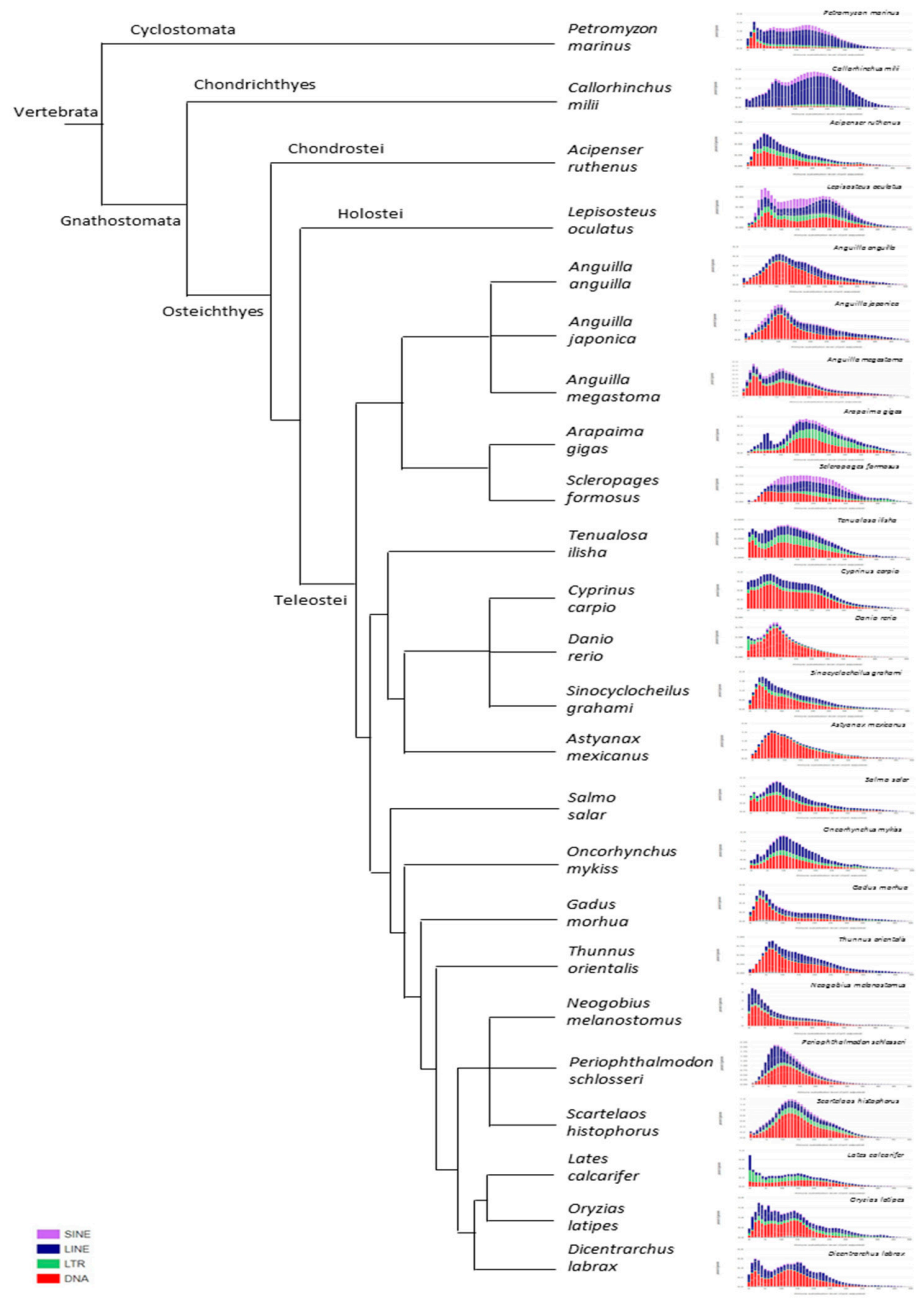
Cladogram with repeat landscape plots. The cladogram reported on the left summarises the phylogenetic relationships among the species analysed in this study. The graphs displayed on the right report the repeat landscape plots obtained by Kimura distance-based copy divergence analyses of transposable elements for each species. The repeat landscape plots are reported at higher resolution in Supplementary File S1.

The analysis of TE sequence divergence by Kimura distance evidenced the presence of one or two amplification bursts in all actinopterygians. The position of these bursts on the left part of the graph (K-value < 25, that indicates a relatively small degree of divergence) suggested a link with recent amplification events. Moreover, the repeat landscapes of the analysed ray-finned fish species showed a remarkable DNA elimination rate due to the distribution of the largest part of TE copies below a K-value of 25 (Figure 4, Supplementary File S1). In most species, DNA transposons represented more than 50% out of all the TEs identified, except for *L. oculatus*, *A. gigas,* and *S. formosus,* in which all the main TE classes were represented. The contribution of SINE retroelements was notable in the spotted gar and in the Asian arowana, in contrast with the other ray-finned fish analysed. No correlation could be observed between the composition of the repeat landscapes and species phylogeny, as clearly summarised in Figure 4.

## 3. Discussion

Genome architecture is one of the key factors influencing the evolutionary success of species [46]. Transposable elements are known to play a pivotal role as a dynamic component of the DNA repetitive fraction. In this framework, the evaluation of the impact of mobile elements in ray-finned fish, one of the most diversified group of vertebrates, may contribute not only to unravel the biodiversity of this taxon, but also to unveil the reason of their extreme adaptability to widely different aquatic environmental conditions. The evolution of specific TE sequences has been linked with environmental temperature [39]. Moreover, an increasing number of studies have reported the additional influence of abiotic factors on TE activity [32,33,37]. Migration is one of the most fascinating behaviours in the lifestyle of some fish species. In particular, this behaviour leads diadromous species to move between environments characterised by substantial differences in abiotic factors (e.g., salinity and temperature). In this study, we investigated the genomes of 15 migratory species: *T. ilisha, O. mykiss,* and *S. salar* are anadromous; *A. anguilla*, *A. japonica,* and *A. megastoma* are catadromous; *N. melanostomus, P. schlosseri,* and *S. histophorus* are amphidromous; *G. morhua, T. orientalis,* and *D. labrax* are oceanodromous; *Acipenser ruthenus, C. carpio,* and *A. mexicanus* are potamodromous. For comparison, two non-bony fish (the jawless *P. marinus* and the elephant shark *C. milii*) and seven additional non-migratory actinopterygian species were considered (Figure 1).

Our results showed a variable TE content both in the bony and non-bony fish genomes analysed, as well as a positive correlation between TE content and the assembled genome size. This suggested, in line with previous reports, that these elements affected the content of nuclear DNA [7,12,22,23,47,48,49,50,51]. In addition, differences in TE content were not associated either with the number of whole genome duplication (WGD) events the analysed species underwent, nor with the ploidy level. In particular, the genome of actinopterygians experienced two rounds of WGDs [52,53,54], and a third round occurred in teleosts [54,55,56]. Several other ray-finned fish species experienced a fourth independent WGD event [3,4,5,57].

In the TE relative contribution analyses, LINE retroelements had the highest impact in sea lamprey and elephant shark, as previously reported by other authors [22,25,40]. Although the known trend of higher relative abundance of DNA transposons in ray-finned fish was confirmed by our analyses, we evidenced some exceptions. In particular, *A. ruthenus, L. oculatus, A. gigas, S. formosus, T. ilisha, O. mykiss, G. morhua, L. calcarifer*, and *O. latipes* showed a lower number of DNA transposons compared with retroelements. The high relative TE content of DNA transposons observed in the only available species of Acipenseriformes, *A. ruthenus*, might reflect a condition similar to that one present in the common ancestor of Actinopterygii.

However, the patterns of TE relative abundance observed across species (Figure 2) were not consistent with phylogeny. This suggested that the accumulation of specific TEs might be related to the evolutionary adaptation of species to specific environmental niches. Indeed, variation partitioning analyses evidenced a statistically significant influence of migratory behaviour on quantitative differences of DNA transposons between catadromous and amphidromous species. The effect of migration was more pronounced on the quantitative difference reported for SINE retroelements in the comparison between anadromous and catadromous fish species. One study has reported that resident and migratory populations of the teleost *Coilia nasus* carry a different SINE copy number. These differences have been related to the anadromous ecotypes of *C. nasus* that cope with environmental challenges during their life cycle [41]. The abundance of SINE retroelements may contribute to the genomic variation in fish, affecting gene expression. Indeed, SINEs are often enriched at the boundaries of transcriptionally active or inactive domains and these elements are thought to be involved in defining high-order genomic organisation through intra- or inter-chromosomal interactions [58,59,60].

The analysis performed to identify fine-scale expansions of SINE retroelements between catadromous and anadromous species showed that most of the elements experienced an expansion in catadromous species. Moreover, the relationships between SINE retroelements patterns observed for the three species of *Anguilla* genus were different from those obtained considering all TEs. These findings strengthen the hypothesis that the similarity in the abundance of SINE retroelements between *Anguilla* species analysed might be also due to different causes from phylogeny. However, the differences in SINE content between catadromous and anadromous species could be investigated more extensively when genomic data from other catadromous species not belonging to *Anguilla* genus are available.

On the contrary, the relative contribution of TEs did not show a clear pattern, either among oceanodromous or among potamodromous species. This finding might be linked to the fact that these species spend their entire life in marine- and freshwaters, respectively, without having to face substantial environmental changes. Overall, the comparison between the oceanodromous–potamodromous and the anadromous–amphidromous–catadromous groups evidenced a lower SINE content in the former, strengthening the hypothesis that these retroelements play a key role in the genomes of fish species characterised by diadromous migratory behaviour. However, further investigations are needed to clarify the cause–effect relationships between the co-evolution of the number of SINE retroelements and the migratory behaviour of fish.

The reconstruction of the transposition history through Kimura distance showed one or two amplification bursts in the repeat landscape profiles of catadromous and anadromous species, with the differences in the relative content of SINE that might be explained by three scenarios. The higher relative content of SINEs in catadromous species is probably related to a higher rate of their transposition. Moreover, purifying selection could have acted as the main driver in determining the discrepancy in TE transposition rate between catadromous and anadromous species. On the other hand, the effect of TE silencing and/or elimination mechanisms might be more active in anadromous species, affecting SINE retroelements content. Moreover, the role played by horizontal transfer events [61,62,63] might not be negligible, because these could have conveyed beneficial SINE retroelements in the host genome of catadromous species, leading to their expansion.

Finally, the importance of sequencing depth and the assembly methods need to be considered, mainly with respect to repeat-rich regions, because these technical factors might influence the estimation of transposable element content. However, the combination of species-specific repeat libraries with the de novo discovery and annotation process described in this work allowed us to improve the identification of previously unidentifiable non-canonical repeats.

## 4. Materials and Methods

A total of 24 fish species were considered in this study: a single species belonging to Cyclostomata (lamprey, *P. marinus*), within Gnathostomata, one member of Chondrichthyes (elephant shark, *C. millii*) and 22 Osteichthyes. These all belong to Actinopterygii and, in detail included one Chondrostei (sterlet, *A. ruthenus*), one Holostei (spotted gar, *L. oculatus*) and 20 Teleostei (European eel, *A. anguilla*; Japanese eel, *A. japonica*; Polynesian longfinned eel, *A. megastoma*; pirarucu, *A. gigas*, arowana, *S. formosus*; Hilsa Shad, *T. ilisha*; carp, *C. carpio*; zebrafish, *D. rerio*; golden line barbel, *S. grahami*; cavefish, *A. mexicanus*; salmon, *S. salar*; rainbow trout, *O. mykiss*; Atlantic cod, *G. morhua*; tuna, *T. orientalis*; round goby, *N. melanostomus*; giant mudskipper, *P. schlosseri*; walking goby, *S. histophorus*; barramundi, *L. calcarifer*; medaka, *O. latipes*; European seabass, *D. labrax*). Unmasked genomes of these species were obtained from the public database NCBI GenBank (https://www.ncbi.nlm.nih.gov/genome/), except for the Atlantic cod genome, which was downloaded from Ensembl genome browser (https://www.ensembl.org/index.html). Accession numbers are reported in Supplementary Table S3.

To identify transposable elements in the analysed species, we used species-specific TE libraries derived from the FishTEDB database (http://www.fishtedb.org/). For the species *A. ruthenus*, *A. gigas*, *T. ilisha*, *C. carpio*, *O. mykiss*, *S. salar*, *N. melanostomus, P. schlosseri,* and *S. histophorus*, de novo TE libraries were built as follows. De novo TE identification was performed using RepeatScout v 1.0.5 [64]: the “build_lmer_table” module generated a table of lmer frequency and “RepeatScout” extracted the repeats that were then filtered with “filter-stage-1.prl” script in order to remove low complexity sequences. The filtered output file was used by RepeatMasker v 4.1.0 (http://www.repeatmasker.org/cgi-bin/WEBRepeatMasker) as a library to extract repeats from each downloaded genome. The RepeatScout “filter-stage-2.prl” script was then employed to remove sequences repeated less than 10 times. The remaining sequences were then filtered by BLASTX [65] search against the Uniprot–Swissprot database [66] and by Interproscan v5.34-73.0 [67], by removing sequences with at least one hit (e-value = 1e-50), because they could be coding sequences not ascribable to TEs. In order to avoid the loss of domesticated transposons sequences filtered out in the previous step, discarded elements were analysed with HMMER [68], searching for domains ascribable to integrase, reverse transcriptase, and transposase functions. The corresponding HMM profiles (PF13333.7, PF13683.7, PF00665.27, PF00078.28, PF13843.7) were downloaded from Pfam [69]. Sequences with an e-value lower than 1e-5 were then reintegrated in the TE library. Finally, TEclass-2.13 was used to classify the remaining sequences. All the libraries were used to mask each genome with RepeatMasker, setting the—a argument in order to obtain the alignment file for each species.

An extended library comprising all TEs identified in catadromous and anadromous fish species was used to mask their genomes, obtaining a comparable dataset among species, representative of the relative genomic abundance of specific TE families. This was calculated by dividing the total amount of masked nucleotides for each family by the total amount of masked nucleotides using the entire library on each genome.

To estimate TE age and transposition history in actinopterygian genomes, Kimura distances (rate of transition and transversions) were calculated between genome total sequence length and TE consensus from the library using the scripts “calcDivergenceFromAlign.pl” and “createRepeatLandscape.pl” provided by the RepeatMasker package.

The main steps of the pipeline are graphically summarised in Supplementary Figure S3.

To test a relationship between genome size (total sequence length) and total TE content (total TE, reported here as a percentage of the total genome size), we applied the Spearman and Kendall rank correlation tests using “cor.test()” function in R.

Variation partitioning analysis was performed using vegan package 2.5.5 [70] to evaluate the influence of transposable elements and/or phylogeny on migratory behaviour. Migration was assigned as the explanatory variable X1 and phylogeny (considered as the sequence divergence obtained by the p-distance matrix using 16S rDNA) as the explanatory variable X2. For each pair of species, both explanatory variables were compared with data related to the difference calculated for each TE type (response variable X).

## 5. Conclusions

The evolutionary success of species is strictly related to the composition and functionality of their genome. Transposable elements undoubtedly have a key role in shaping genome architecture by generating the genetic innovations responsible for species’ adaptability. Ray-finned fish are characterised by an extremely high diversity and are adapted to a wide range of environments. The data presented here did not show any significant correlation between TE composition and the phylogenetic relationships among species, although they indicated an interesting link between the genomic content of mobile elements and environmental conditions. It is not easy to establish whether the content of a specific TE-type is determined by the environment in which a given species lives or vice versa. However, these findings follow the well-established trends that envision TEs as primary drivers of the exceptional biodiversity of species and lay the foundation for further experimental analyses towards the comprehension of mechanisms and factors involved in this fascinating relationship.

## Supplementary Materials

Supplementary materials can be found at https://www.mdpi.com/1422-0067/22/2/602/s1.
