# Supplementary material for: Transposable Elements and Teleost Migratory Behaviour
Source: Int J Mol Sci. 2021 Jan 9;22(2):602. doi: 10.3390/ijms22020602 (PMC7827017; doi:10.3390/ijms22020602)

Petromyzon marinus

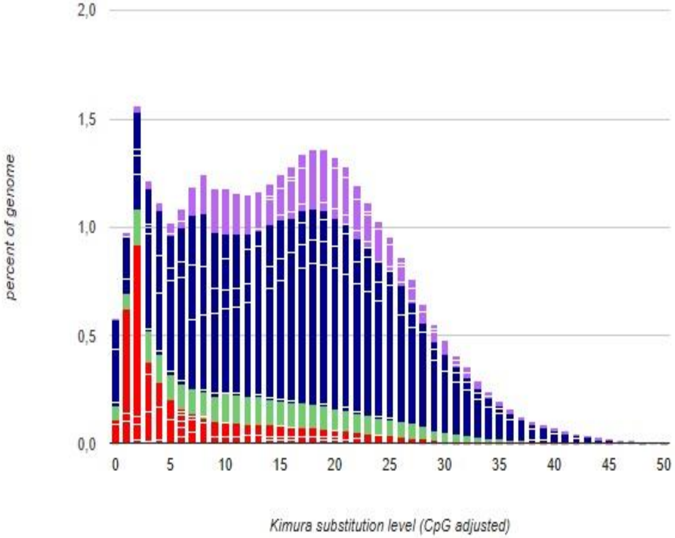

Callorhinchus milii

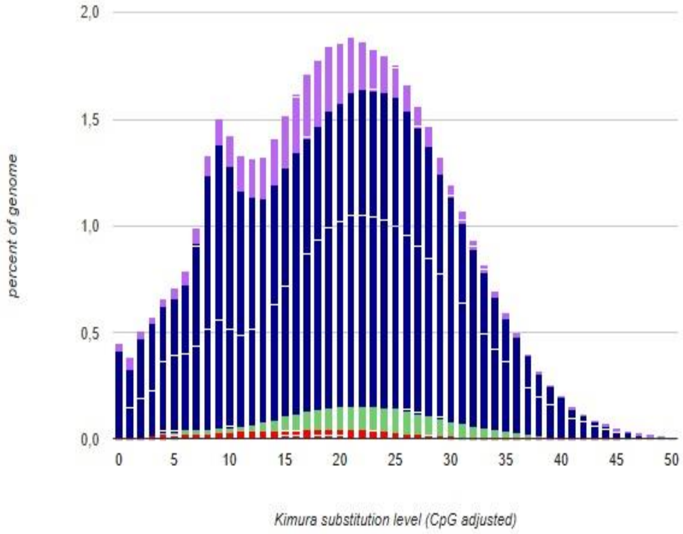

Acipenser ruthenus

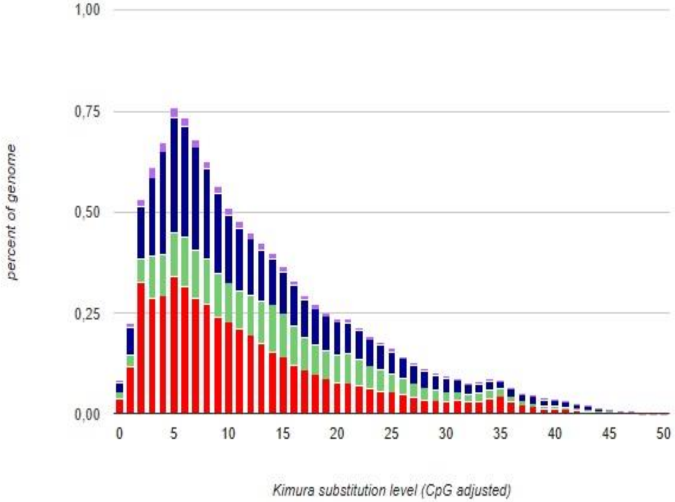

Lepisosteus oculatus

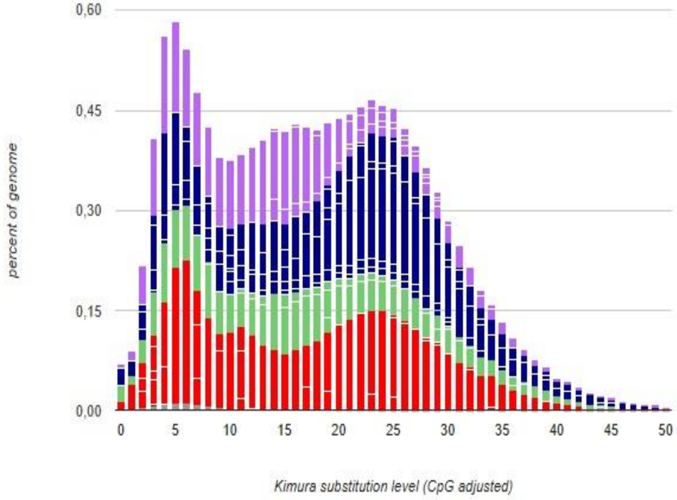

Anguilla anguilla

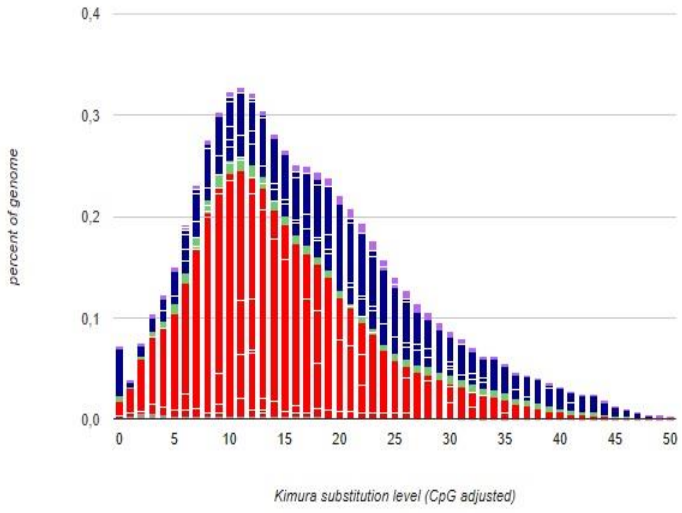

Anguilla japonica

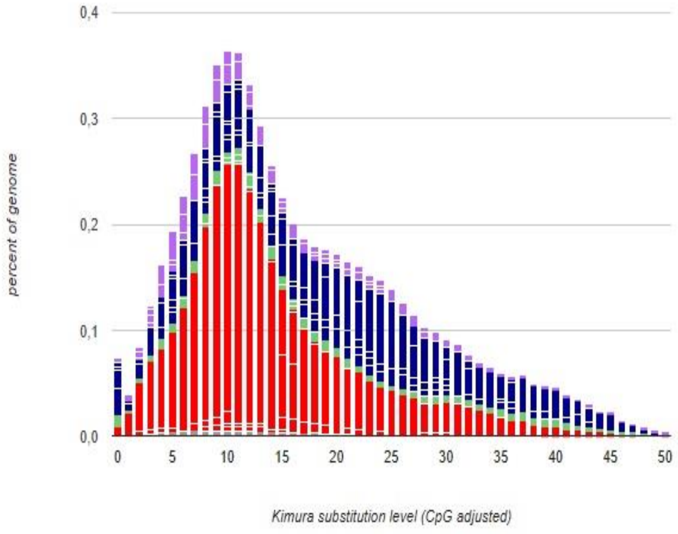

Anguilla megastoma

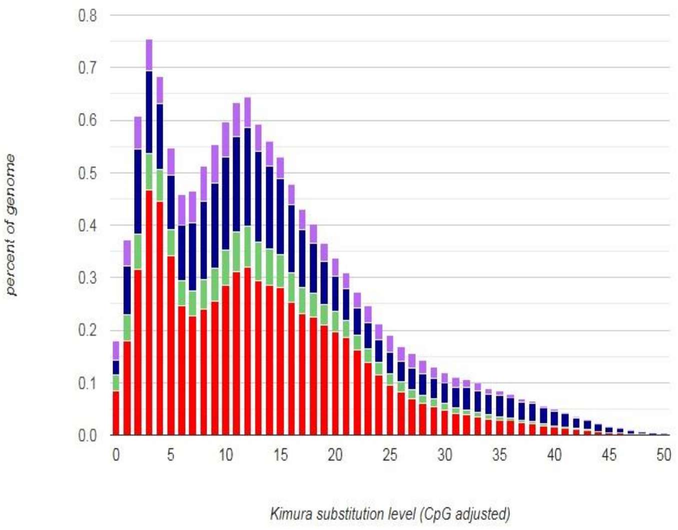

Arapaima gigas

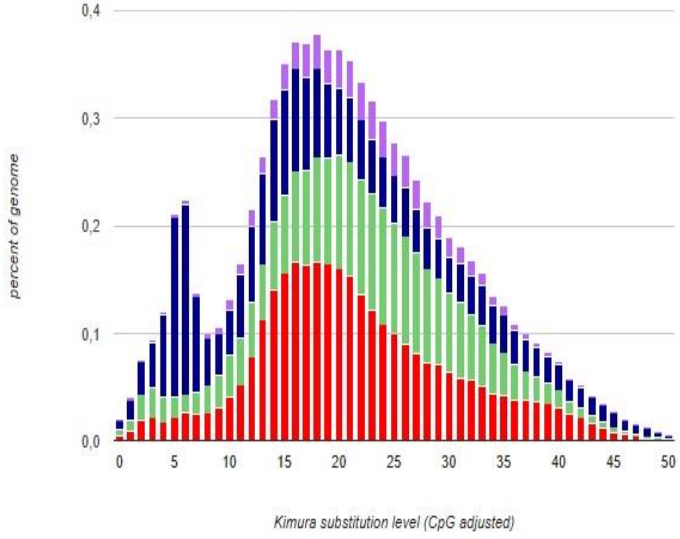

Scleropages formosus

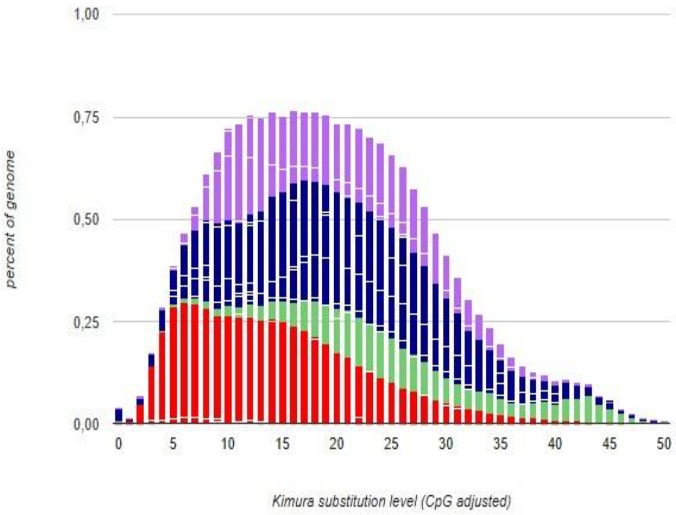

Tenualosa ilisha

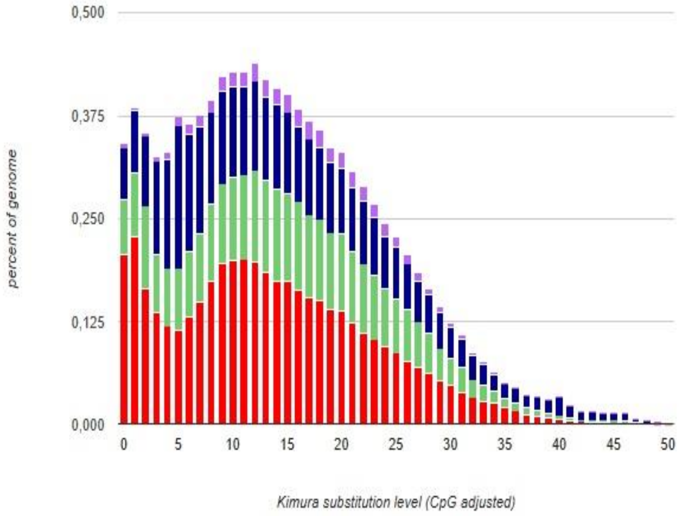

Cyprinus carpio

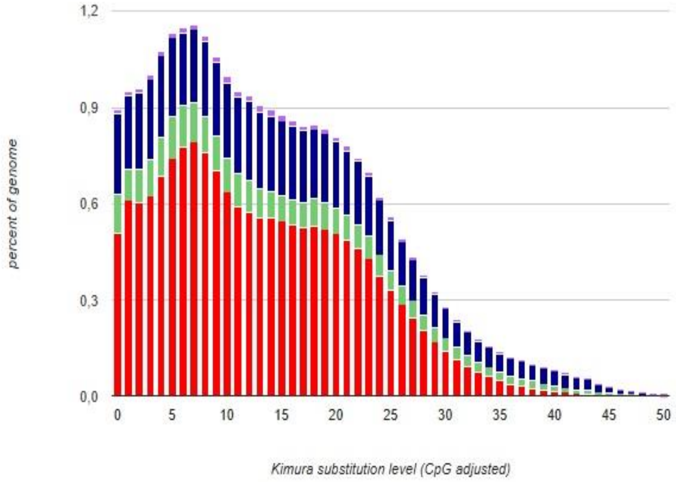

Danio rerio

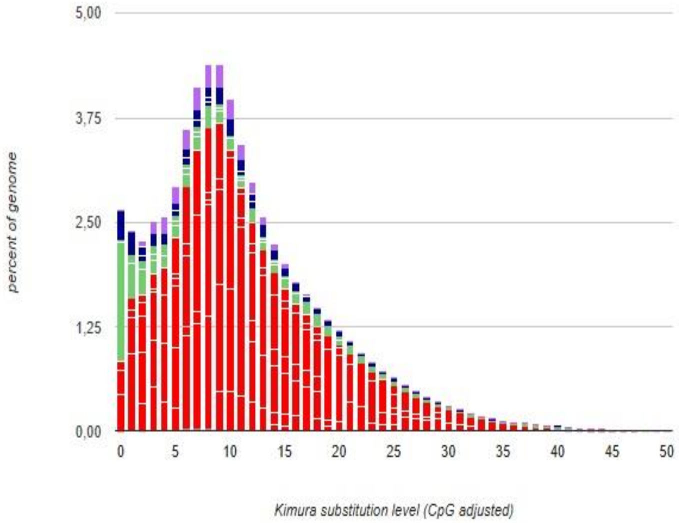

Sinocyclocheilus grahami

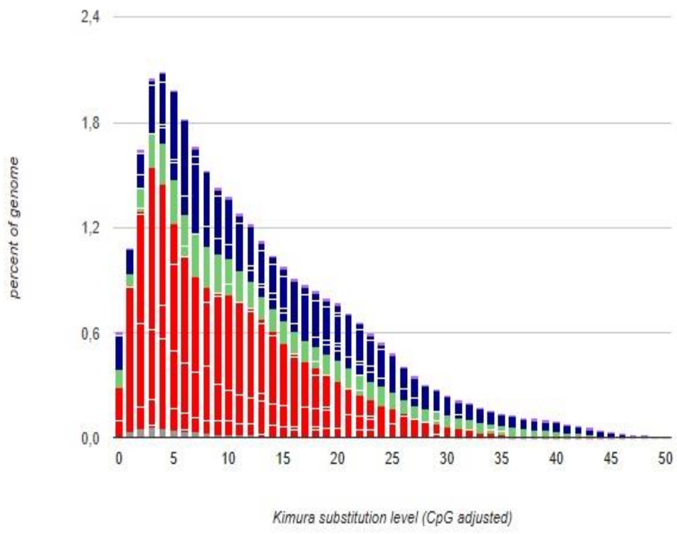

Astyanax mexicanus

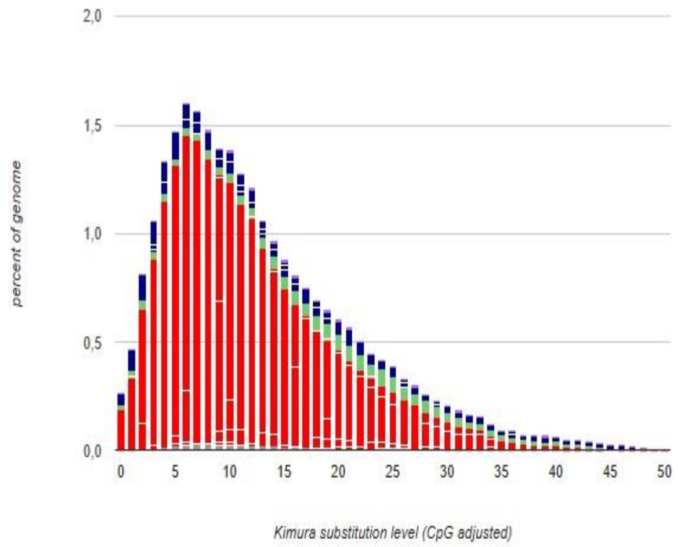

Salmo salar

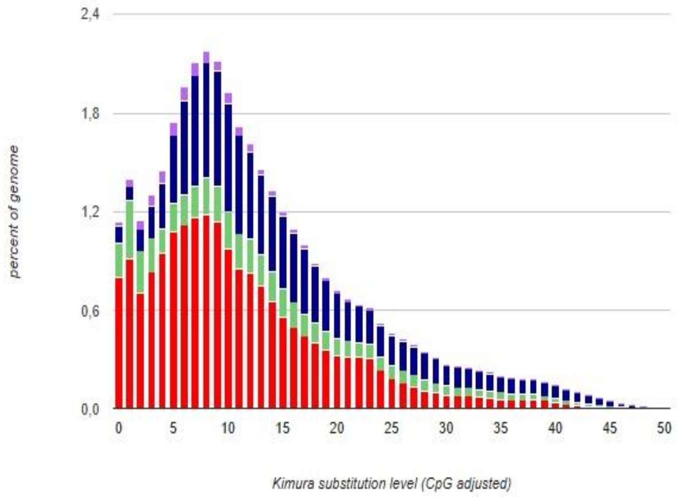

Oncorhynchus mykiss

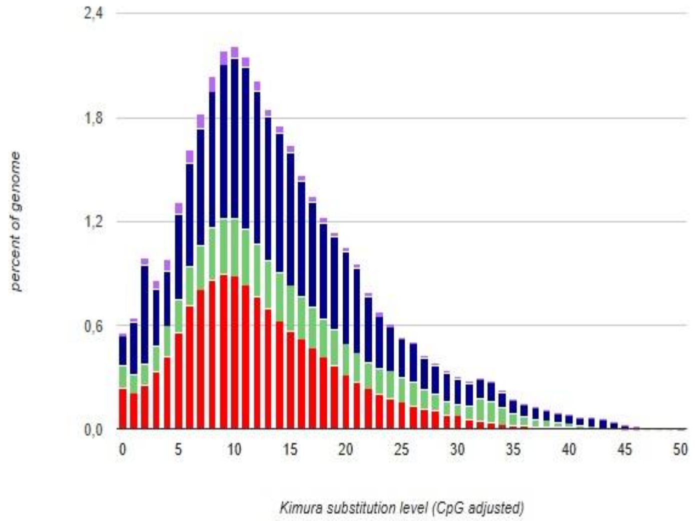

Gadus morhua

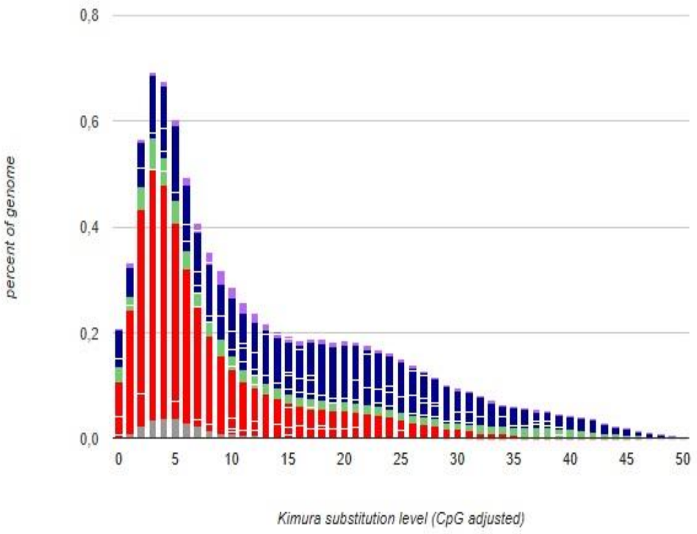

Thunnus orientalis

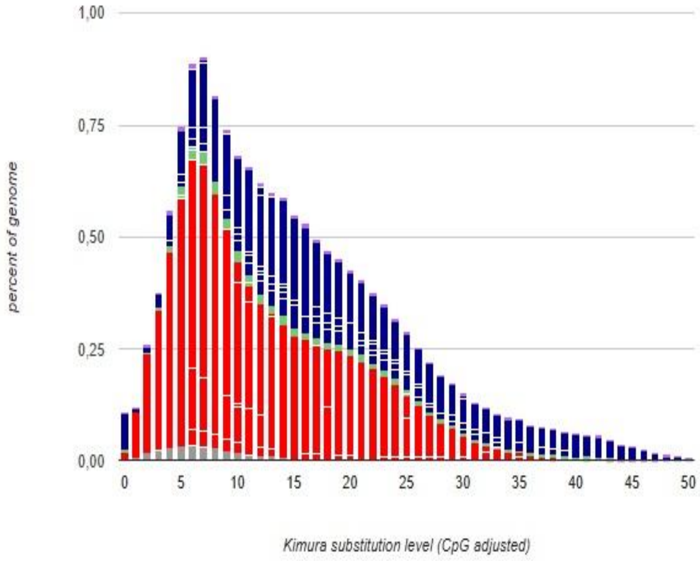

Neogobius melanostomus

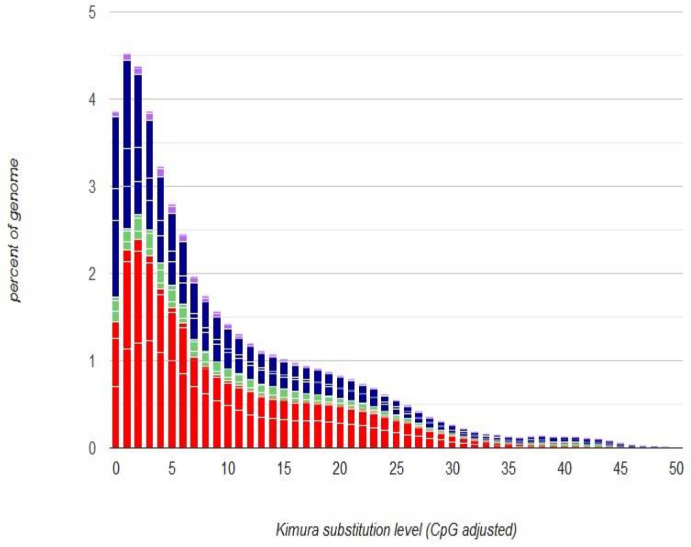

Periophthalmodon schlosseri

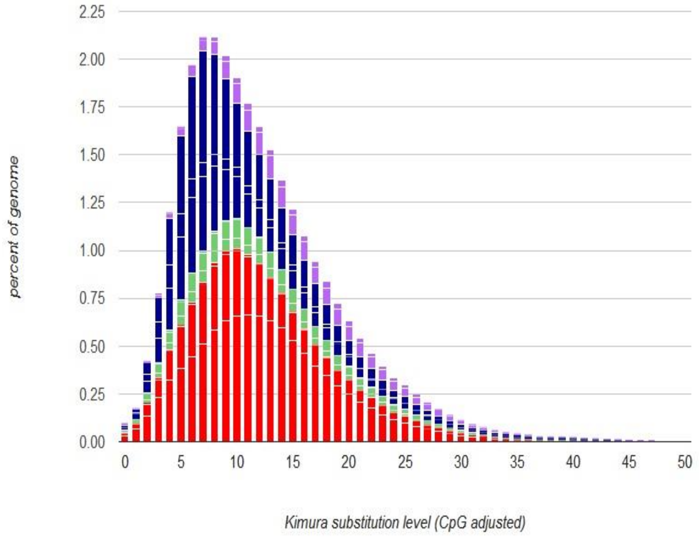

Scartelaos histophorus

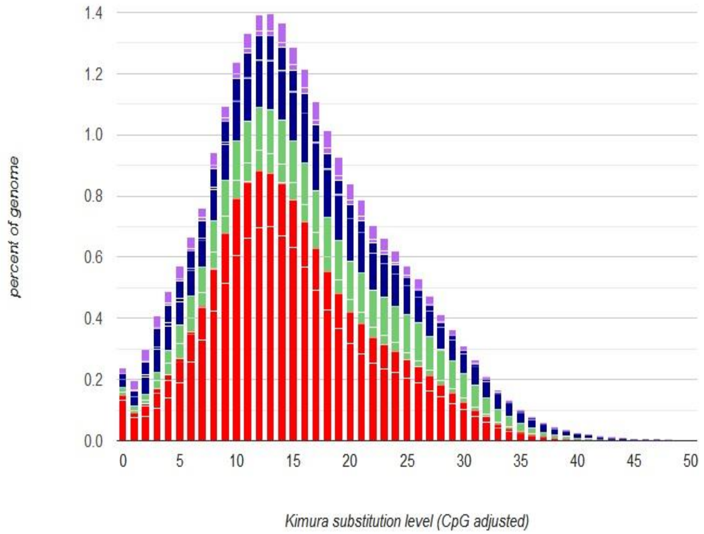

Lates calcarifer

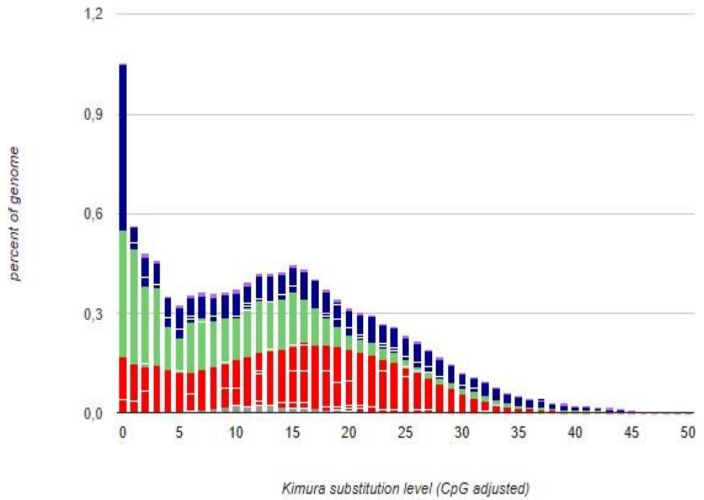

Oryzias latipes

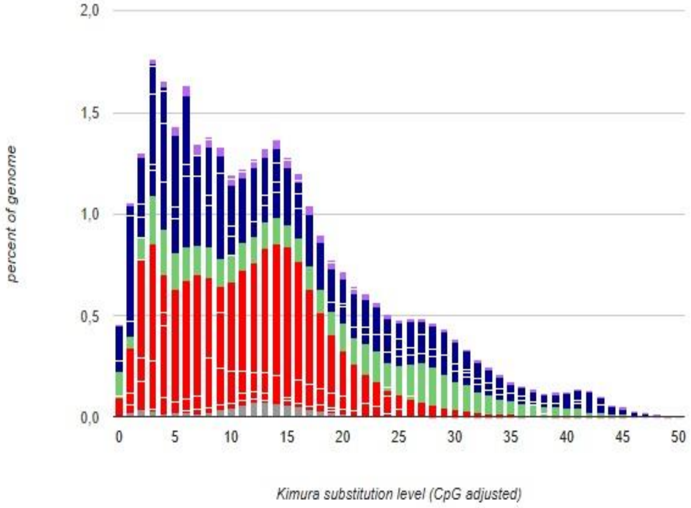

Dicentrarchus labrax

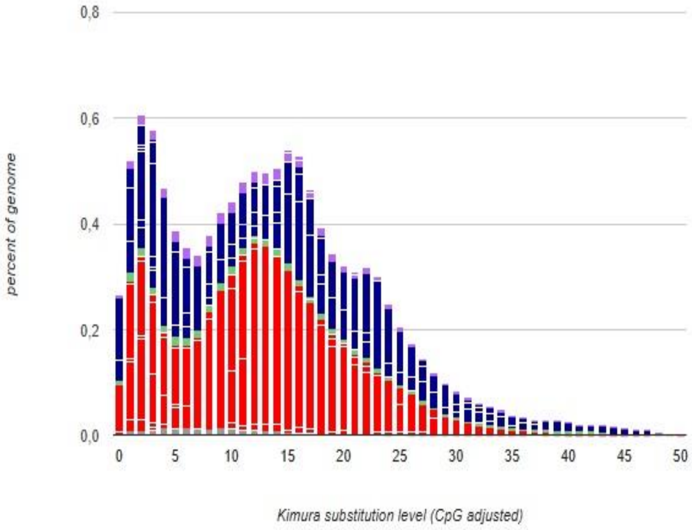

Supplement: Supplementary file 1 [file ijms-22-00602-s001.zip › Supplementary_material/SupplementaryFileS1.pdf]
