# Supplementary material for: Transposable Elements and Teleost Migratory Behaviour
Source: Int J Mol Sci. 2021 Jan 9;22(2):602. doi: 10.3390/ijms22020602 (PMC7827017; doi:10.3390/ijms22020602)
Supplement: Supplementary file 1 [file ijms-22-00602-s001.zip › Supplementary_material/SupplementaryTables/TableS1.docx]

| **SPECIES** | **LINE %** | **SINE %** | **LTR %** | **DNAtranspon %** | **Unclassified %** | **TOT_TE %** | **Tot_Seq_Length** |
| --- | --- | --- | --- | --- | --- | --- | --- |
| *Petromyzon marinus* | 28.94 | 7.23 | 12.06 | 5.58 | 2.18 | 55.99 | 885534757 |
| *Callorhinchus milii* | 33.43 | 5.53 | 3.3 | 0.67 | 2.30 | 45.23 | 974498586 |
| *Acipenser ruthenus* | 7.89 | 1.03 | 5.17 | 10.6 | 6.01 | 30.70 | 1732545901 |
| *Lepisosteus oculatus* | 5.35 | 2.83 | 2.89 | 4.00 | 1.06 | 16.13 | 945878036 |
| *Anguilla anguilla* | 2.98 | 0.93 | 1.4 | 5.63 | 1.78 | 12.72 | 1018701900 |
| *Anguilla japonica* | 3.95 | 1.26 | 1.51 | 6.99 | 1.74 | 15.45 | 1151137423 |
| *Anguilla megastoma* | 3.37 | 1.43 | 1.43 | 6.19 | 3.04 | 15.46 | 877765645 |
| *Arapaima gigas* | 2.23 | 0.58 | 2.28 | 2.77 | 1.75 | 9.61 | 661278939 |
| *Scleropages formosus* | 6.96 | 4.88 | 2.91 | 5.49 | 0.21 | 20.45 | 746544453 |
| *Tenualosa ilisha* | 2.83 | 0.44 | 2.57 | 4.26 | 5.29 | 15.39 | 815647530 |
| *Cyprinus carpio* | 6.2 | 0.44 | 2.87 | 14.87 | 6.07 | 30.45 | 1713658011 |
| *Denio rerio* | 3.25 | 2.99 | 5.87 | 44.29 | 0.80 | 57.20 | 1373454788 |
| *Synocyclocheilus grahami* | 10.26 | 0.27 | 5.49 | 16.93 | 1.20 | 34.15 | 1750287761 |
| *Astyanax mexicanus* | 4.85 | 0.32 | 2.78 | 21.46 | 2.19 | 31.60 | 1335239194 |
| *Oncorhynchus mykiss* | 13.49 | 1.12 | 6.03 | 12.18 | 9.54 | 42.36 | 2178999613 |
| *Salmo salar* | 10.13 | 0.98 | 4.83 | 16.23 | 7.41 | 39.58 | 2966890203 |
| *Gadus morhua* | 4.86 | 0.34 | 1.85 | 4.19 | 1.32 | 12.56 | 832114588 |
| *Thunnus orientalis* | 6.72 | 0.38 | 1.77 | 8.65 | 1.50 | 19.02 | 786596543 |
| *Neogobius melanostomus* | 15.63 | 1.23 | 4.19 | 24.08 | 7.44 | 45.13 | 1003738541 |
| *Periophthalmodon schlosseri* | 9.57 | 1.64 | 2.69 | 13.56 | 8.08 | 27.46 | 679761122 |
| *Scartelaos histophorus* | 4.75 | 1.55 | 3.82 | 12.25 | 6.67 | 22.37 | 695008792 |
| *Lates calcarifer* | 2.76 | 0.26 | 5.54 | 4.18 | 0.50 | 13.24 | 589095062 |
| *Oryzias latipes* | 11.91 | 1.15 | 6.47 | 14.30 | 1.70 | 35.53 | 734057086 |
| *Dicentrarchus labrax* | 4.69 | 0.62 | 0.99 | 6.21 | 1.21 | 13.72 | 675917103 |

**Supplementary Table S1.** List of species considered in this study and pencentages of each TE type and global TE content obtained from genome masking analysis. In the last column the total sequences length is shown.
