# Supplementary material for: Transposable Elements and Teleost Migratory Behaviour
Source: Int J Mol Sci. 2021 Jan 9;22(2):602. doi: 10.3390/ijms22020602 (PMC7827017; doi:10.3390/ijms22020602)
Supplement: Supplementary file 1 [file ijms-22-00602-s001.zip › Supplementary_material/SupplementaryTables/TableS2.docx]

|  | **SPECIES** | **relative LINE** | **relative SINE** | **relative LTR** | **relative DNA transposons** |
| --- | --- | --- | --- | --- | --- |
| **Catadromous** | *A. anguilla* | 27,24 | 8,50 | 12,80 | 51,46 |
|  | *A. japonica* | 28,81 | 9,19 | 11,01 | 50,98 |
|  | *A. megastoma* | 27,13 | 11,51 | 11,51 | 49,84 |
| **Anadromous** | *T. ilisha* | 28,02 | 4,36 | 25,45 | 42,18 |
|  | *O. mykiss* | 41,1 | 3,41 | 18,37 | 37,11 |
|  | *S. salar* | 31,49 | 3,05 | 15,01 | 50,45 |
| **Amphidromous** | *S. histophorus* | 30,25 | 9,87 | 24,33 | 78,03 |
|  | *P. schlosseri* | 49,38 | 8,46 | 13,88 | 69,97 |
|  | *N. melanostomus* | 41,47 | 3,26 | 11,12 | 63,89 |

**Supplementary Table S2**. Relative abundance of TE types in the mobilome of diadromy fish species.
