# Supplementary material for: Transposable Elements and Teleost Migratory Behaviour
Source: Int J Mol Sci. 2021 Jan 9;22(2):602. doi: 10.3390/ijms22020602 (PMC7827017; doi:10.3390/ijms22020602)
Supplement: Supplementary file 1 [file ijms-22-00602-s001.zip › Supplementary_material/SupplementaryTables/TableS3.docx]

| **SPECIES** | **ACCESSION NUMBERS** |
| --- | --- |
| *Petromyzon marinus* | GCA_000148955.1 |
| *Callorhinchus milii* | GCA_000165045.2 |
| *Acipenser ruthenus* | GCA_004119895.1 |
| *Lepisosteus oculatus* | GCA_000242695.1 |
| *Anguilla anguilla* | GCA_000695075.1 |
| *Anguilla japonica* | GCA_000470695.1 |
| *Anguilla megastoma* | GCA_901111305.1 |
| *Arapaima gigas* | GCA_900497675.1 |
| *Scleropages formosus* | GCA_001624245.1 |
| *Tenualosa ilisha* | GCA_003651195.1 |
| *Cyprinus carpio* | GCA_000951615.2 |
| *Denio rerio* | GCA_000002035.4 |
| *Synocyclocheilus grahami* | GCA_001515645.1 |
| *Astyanax mexicanus* | GCA_000372685.2 |
| *Oncorhynchus mykiss* | GCA_002163495.1 |
| *Salmo salar* | GCA_000233375.4 |
| *Gadus morhua* | http://www.ensembl.org/Gadus_morhua/Info/Index |
| *Thunnus orientalis* | GCA_009176245.1 |
| *Neogobius melanostomus* | GCA_007210695.1 |
| *Periophthalmodon schlosseri* | GCA_000787095.1 |
| *Scartelaos histophorus* | GCA_000787155.1 |
| *Lates calcarifer* | GCA_001010145.1 |
| *Oryzias latipes* | GCA_002234675.1 |
| *Dicentrarchus labrax* | GCA_000689215.1 |
|  |  |
|  |  |
|  |  |

**Supplementary Table S3**. List of species considered in this study and related accession numbers of genome assembly.
