# Supplementary figures and images for: Transposable Elements and Teleost Migratory Behaviour
Source: Int J Mol Sci. 2021 Jan 9;22(2):602. doi: 10.3390/ijms22020602 (PMC7827017; doi:10.3390/ijms22020602)

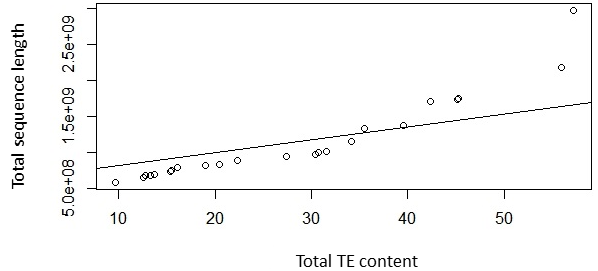

Supplement: Supplementary file 1 [file ijms-22-00602-s001.zip › Supplementary_material/SupplementaryFigure/FigureS1.TIF]

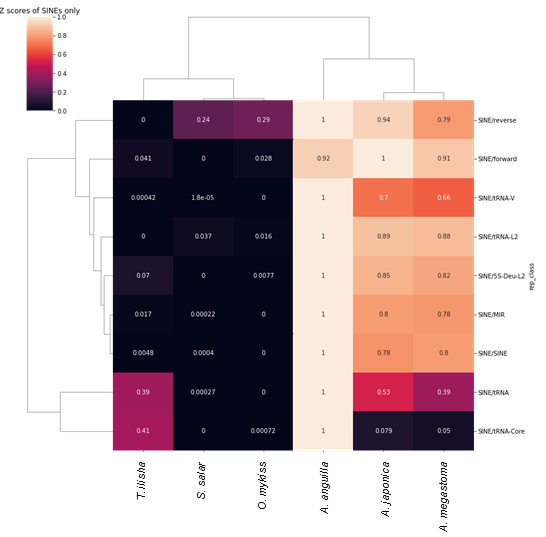

Supplement: Supplementary file 1 [file ijms-22-00602-s001.zip › Supplementary_material/SupplementaryFigure/FigureS2A.tif]

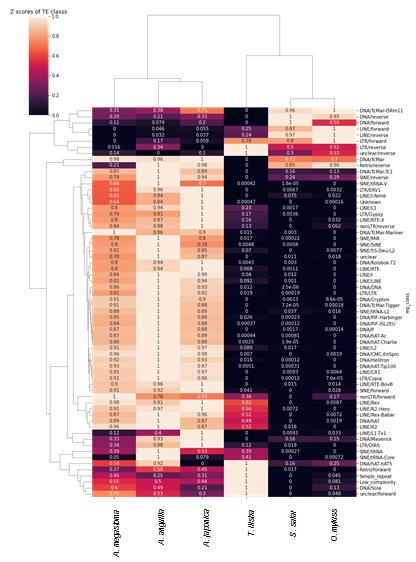

Supplement: Supplementary file 1 [file ijms-22-00602-s001.zip › Supplementary_material/SupplementaryFigure/FigureS2B.tif]

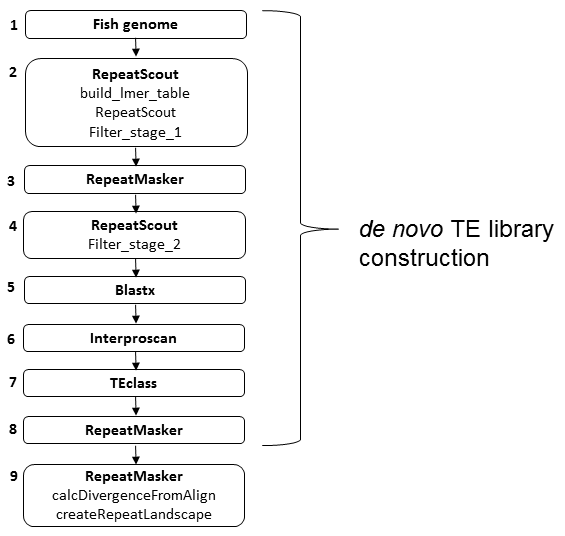

Supplement: Supplementary file 1 [file ijms-22-00602-s001.zip › Supplementary_material/SupplementaryFigure/FigureS3.TIF]
